# Beyond the Obstruction: Recognising Polyneuropathy, Organomegaly, Endocrinopathy, Monoclonal Plasma Cell Disorder, and Skin Changes (POEMS) Syndrome in a Patient With Budd-Chiari Syndrome

**DOI:** 10.7759/cureus.96014

**Published:** 2025-11-03

**Authors:** Shadman Sakib Rahman, Kh Imranul Alam, Nusrat A Chowdhury, Usman Ul Haq, Md Al Amin Sarkar, Sumaiya Kamal, James Alegbeleye

**Affiliations:** 1 Internal Medicine, Medway NHS Foundation Trust, Kent, GBR; 2 Internal Medicine, Medway Maritime Hospital, Gillingham, GBR; 3 General Internal Medicine, Medway NHS Foundation Trust, Kent, GBR; 4 Medicine, Medway Maritime Hospital, Gillingham, GBR; 5 Acute Medicine/General Internal Medicine, Medway NHS Foundation Trust, Kent, GBR; 6 Acute Medicine, Medway Maritime Hospital, Gillingham, GBR; 7 General Medicine, Medway NHS Foundation Trust, Kent, GBR

**Keywords:** anticoagulation, autologous stem cell transplant, budd-chiari syndrome, daratumumab, hepatic vein thrombosis, osteosclerotic lesions, paraneoplastic syndromes, plasma cell, poems syndrome, vegf angiogenesis

## Abstract

Polyneuropathy, organomegaly, endocrinopathy, monoclonal plasma cell disorder, and skin changes (POEMS) syndrome is a rare paraneoplastic disorder caused by an underlying monoclonal plasma cell neoplasm. We report a rare case of a 52-year-old man who presented with subacute sensorimotor neuropathy, hepatosplenomegaly, ascites, pleural effusion, thrombocytosis, gynecomastia, and osteosclerotic bone lesions. Investigations revealed elevated vascular endothelial growth factor (VEGF) levels and clonal plasma cells, consistent with POEMS syndrome. He was also found to have hepatic vein thrombosis, confirming Budd-Chiari syndrome. Treatment with daratumumab, bortezomib, and dexamethasone, along with anticoagulation, led to good symptom control. This case highlights the diagnostic challenges and multisystem presentation of POEMS syndrome. Early identification and multidisciplinary management can help prevent irreversible complications.

## Introduction

Polyneuropathy, organomegaly, endocrinopathy, monoclonal plasma cell disorder, and skin changes (POEMS) syndrome is a paraneoplastic condition driven by an underlying plasma cell dyscrasia [1]. Major diagnostic criteria include polyradiculoneuropathy, clonal plasma cell disorder (PCD), sclerotic bone lesions, elevated vascular endothelial growth factor (VEGF), and Castleman disease, while minor criteria encompass organomegaly, endocrinopathy, characteristic skin changes, papilloedema, extravascular fluid overload, and thrombocytosis [1]. The diagnosis is established when at least three major criteria are met, including both polyradiculoneuropathy and clonal PCD, along with at least one minor criterion [1].

POEMS syndrome is extremely rare, with an estimated prevalence of less than 1 per 1,000,000 individuals [2]. It predominantly affects middle-aged adults, typically in the fourth to sixth decades of life, with a slight male predominance [3].

## Case presentation

A 52-year-old man with no significant past medical history presented with progressive weakness, tingling, and numbness of the lower limbs for the past three months. He also reported significant weight loss of approximately 10 kg over two months, accompanied by worsening fatigue. His mobility gradually declined, eventually requiring the use of a walking frame. Over the past six weeks, he developed increasing abdominal girth with intermittent abdominal pain, bilateral lower limb swelling, and exertional dyspnoea. There was no history of fever, night sweats, or overt bleeding. He also reported altered bowel habits. He was a non-smoker and consumed alcohol only occasionally.

On examination, there was marked abdominal distension with positive shifting dullness on percussion, indicating the presence of ascites. The spleen was palpable, consistent with splenomegaly. Neurological examination revealed bilateral foot drop, reduced lower limb strength (3/5 hip flexors, 4/5 hip extensors, 3/5 knee flexors, 2/5 ankle dorsiflexors), and diminished proprioception below the knees. All reflexes were intact, and bowel and bladder functions were normal. Cranial nerves were intact. He also had mild bilateral gynaecomastia, palmar hyperpigmentation, and bilateral pitting oedema extending up to the mid-thighs. There was no lymphadenopathy or other signs of infection.

These findings indicated multisystem involvement, including neurological (polyneuropathy), endocrine (gynaecomastia), fluid overload (oedema, ascites), organomegaly (splenomegaly), and skin changes (hyperpigmentation). The combination of peripheral neuropathy, organomegaly, endocrinopathy, skin changes, and fluid overload supported a possible diagnosis of POEMS syndrome. Based on these multisystem findings, a clinical suspicion of POEMS syndrome prompted further diagnostic investigations in addition to routine blood tests, the results of which are summarised in Table 1.

**Table 1:** Routine blood and diagnostic investigations CRP: C-reactive protein, TSH: thyroid-stimulating hormone, ESR: erythrocyte sedimentation rate, ALP: alkaline phosphatase, ALT: alanine aminotransferase, VEGF: vascular endothelial growth factor, ANA: antinuclear antibody, ANCA: anti-neutrophil cytoplasmic antibody.

Investigations	Results	Normal values
Haemoglobin (g/L)	138	130-170
Platelet count (x10^9^)	487	150-450
Creatinine (µmol/L)	132	59-104
Urea (mmol)	10.4	2.5-7.8
Potassium (mmol/L)	5.5	3.5-5.3
Sodium (mmol/L)	137	133-146
CRP (mg/L)	28.9	5
TSH (mU/L)	6.34	0.4-4.0
Free T4 (pmol/L)	9.4	12-22
ESR (mm/h)	26	0-10
Albumin (g/L)	37	35-50
ALP (U/L)	120	30-130
ALT (U/L)	15	7-56
Total bilirubin (µmol/L)	8	0-21
VEGF (pg/mL)	5996	<600
Urine Bence Jones protein	Negative	
ANA, ANCA, and serum autoimmune profile	Negative	
Serum protein electrophoresis	Mild polyclonal hypergammaglobulinaemia. Serum free light chains: kappa 88 mg/L, lambda 72.6 mg/L, with a kappa-to-lambda ratio of 1:2	

**Figure 1:**
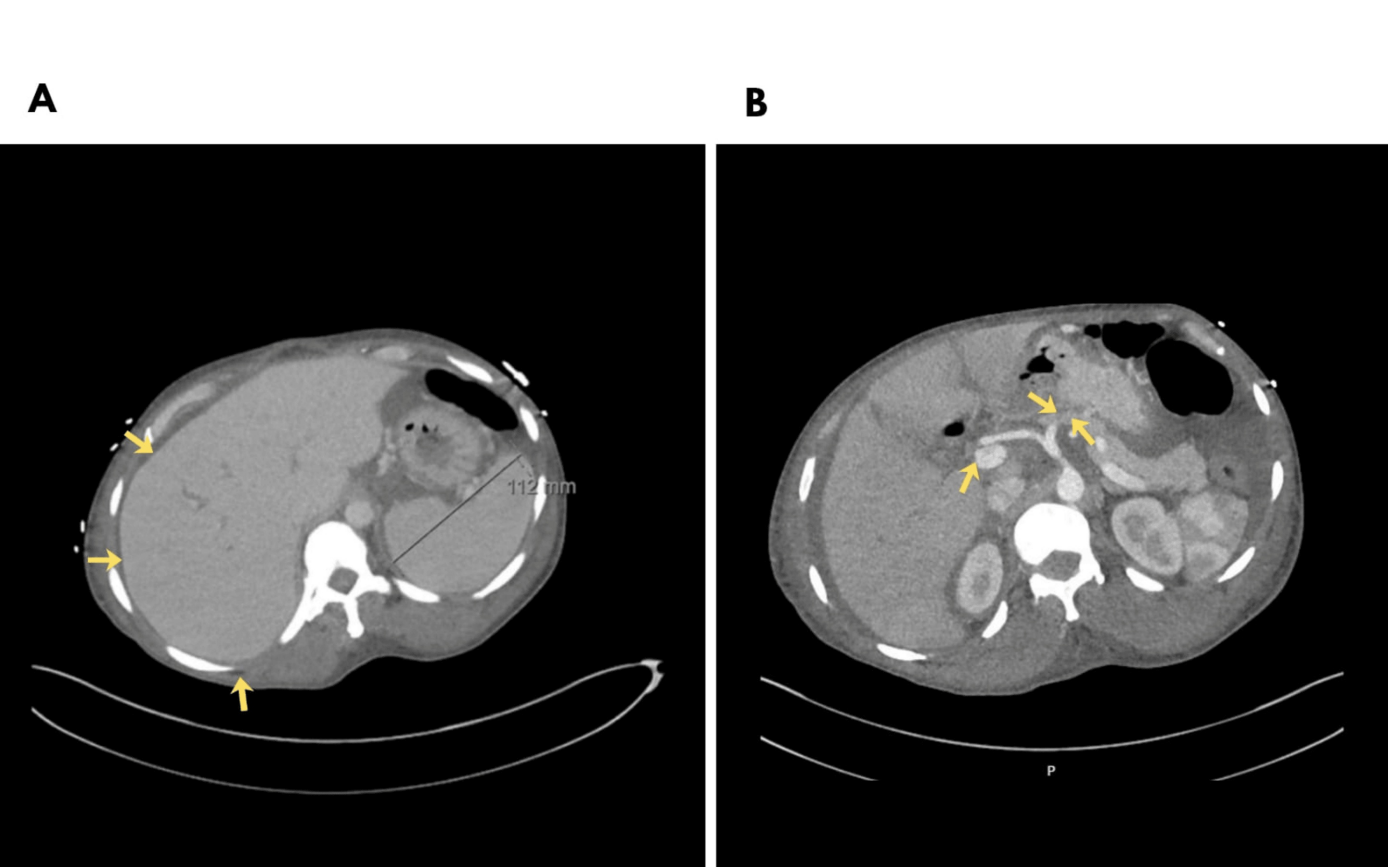
Axial sections of the triple-phase liver CT showing (A) splenomegaly (12.9 cm) with hepatic congestion and (B) thrombus within the hepatic veins CT: computed tomography.

**Figure 2:**
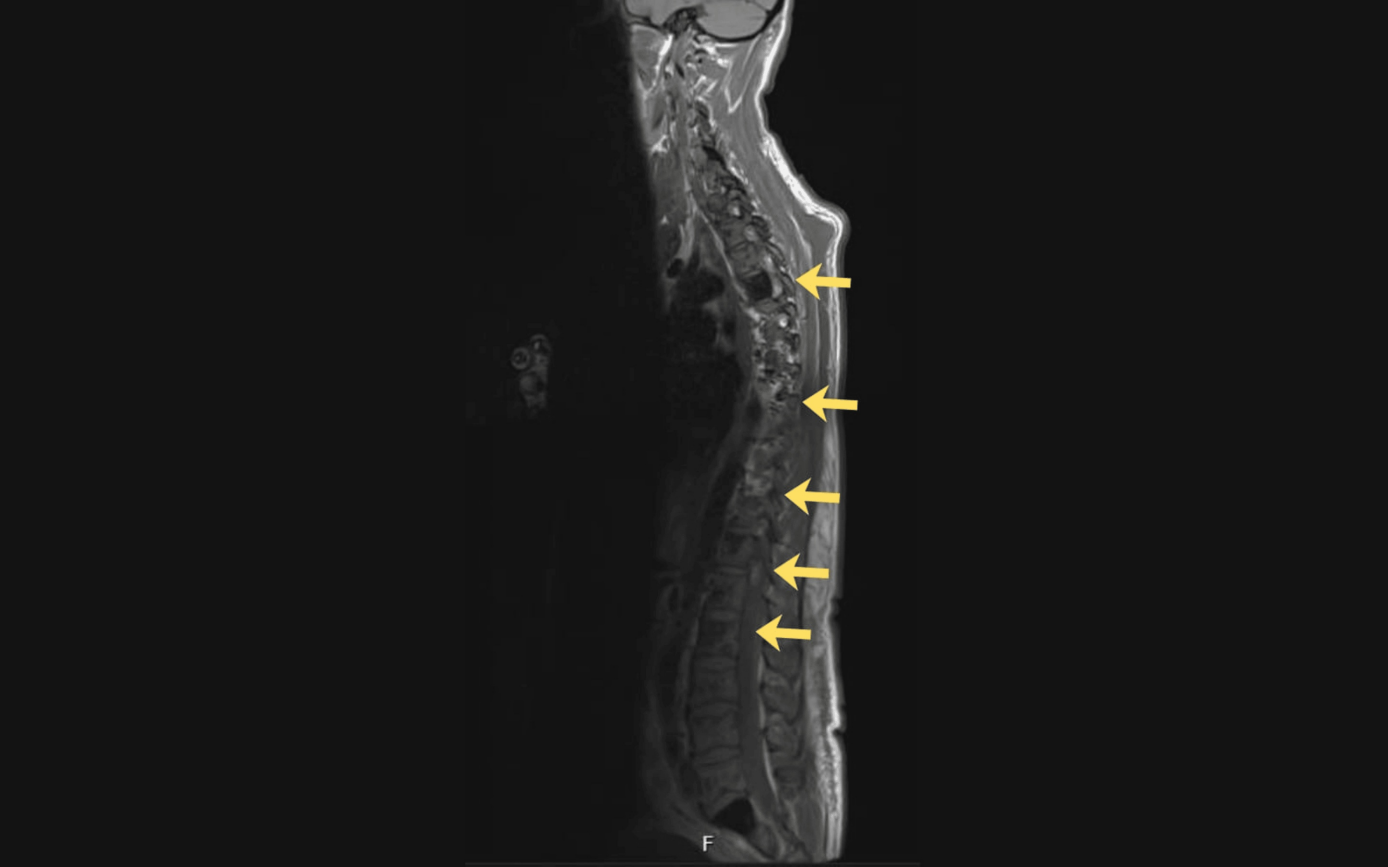
Whole-spine MRI (sagittal section) showing multiple sclerotic lesions in the vertebrae and visualised bones (arrows), with degenerative disc changes from C3 to C7 MRI: magnetic resonance imaging, C3-C7: the third through seventh cervical vertebrae.

A diagnostic ascitic tap was performed, and the fluid was found to be transudative in nature with no malignant cells. Multiple imaging studies, including CT of the thorax, abdomen, and pelvis, triple-phase CT of the liver (Figure 1), MRI of the whole spine (Figure 2), bone scan, and PET-CT, were conducted. Nerve conduction studies and biopsies from the liver and bone marrow were also undertaken, and the results are summarised in Table 2.

**Table 2:** Summary of imaging modalities and biopsy findings demonstrating multisystem involvement in POEMS syndrome CT: computed tomography, MRI: magnetic resonance imaging, PET-CT: positron emission tomography-computed tomography.

Investigations	Results
Abdominal and pelvic CT	Gross ascites, hepatic vein thrombosis (suggestive of Budd–Chiari syndrome), splenomegaly, and widespread osteosclerotic bone lesions in the spine and pelvis
Triple-phase liver CT	Extensive hepatic vein thrombus with evidence of hepatic congestion. Splenomegaly (12.9 cm). Thrombus is present throughout the middle and left hepatic veins; the right hepatic vein is patent centrally, but its peripheral branches contain thrombus
Chest CT	Bilateral pleural effusions and scattered sclerotic bone lesions
Whole-spine MRI	Multiple sclerotic lesions in the spine and visualised bones. Degenerative disc changes from C3 to C7 without significant canal stenosis or nerve root compression
Bone scan	Mildly increased uptake in the knees and sacroiliac joints
PET-CT	Widespread non-FDG-avid sclerotic bone lesions; no overt malignancy
Nerve conduction study (lower limb)	Demyelinating sensorimotor polyneuropathy
Bone marrow biopsy	Approximately 10% clonal plasma cells with associated megakaryocytic hyperplasia and focal sclerotic changes. Immunohistochemistry: CD138-positive with light chain restriction
Liver biopsy	Nodular regenerative hyperplasia consistent with chronic hepatic outflow obstruction, with no evidence of cirrhosis or malignancy

The diagnosis of POEMS syndrome in this patient was confirmed based on the fulfillment of the mandatory, major, and minor criteria outlined in Table 3. The combination of characteristic clinical, radiological, and laboratory findings supports the diagnosis and is consistent with established criteria for this rare multisystem disorder.

**Table 3:** Diagnostic criteria for POEMS syndrome identified in the patient POEMS: polyneuropathy, organomegaly, endocrinopathy, monoclonal plasma cell disorder, and skin changes.

Criterion	Clinical features
Mandatory criteria	Demyelinating polyneuropathy; monoclonal plasma cell disorder (bone marrow involvement)
Major criteria	Osteosclerotic bone lesions; elevated VEGF (>5000 pg/mL)
Minor criteria	Organomegaly (splenomegaly); endocrinopathy (subclinical hypothyroidism, gynecomastia); extravascular fluid overload (ascites, pleural effusions); skin changes (palmar hyperpigmentation); thrombocytosis

He also had Budd-Chiari syndrome, confirmed by imaging and liver biopsy, likely due to the prothrombotic state associated with POEMS.

Management

This complex multisystem diagnosis was discussed in a multidisciplinary team meeting, which included Hepatology, Neurology, Endocrinology, Haematology, Physiotherapy, and Occupational Therapy.

Treatment was commenced with daratumumab, bortezomib, and dexamethasone (Dara-Vd), following advice from the Haematology team. Thalidomide was avoided due to hepatic vein thrombosis. For anticoagulation, the patient was initially treated with therapeutic low-molecular-weight heparin (enoxaparin) and was later switched to warfarin. Management of ascites included fluid restriction and spironolactone (40 mg twice daily), as recommended by the Hepatology team, with ongoing follow-up in their clinic. Hepatitis B prophylaxis was initiated with entecavir. Following Endocrinology advice, thyroid hormone replacement therapy was also started. Physiotherapy was initiated early to support mobility and rehabilitation.

Follow-up

Following initiation of chemotherapy, the patient’s ascites and pleural effusions gradually improved. Neurological symptoms stabilised, although full motor recovery was not observed during the initial phase of treatment. Repeat VEGF testing is planned at six weeks to monitor trends, and CT imaging at three months is scheduled to reassess hepatic vein patency. He remains on long-term anticoagulation and entecavir and continues routine physiotherapy. At discharge, he was able to transfer independently with aids and perform limited self-care. He was referred to a regional Haematology unit for consideration of autologous stem cell transplantation.

## Discussion

POEMS syndrome is an uncommon, multisystem paraneoplastic disorder secondary to an associated monoclonal plasma cell neoplasm [2,3]. It is distinct from classical multiple myeloma, as it is associated with sclerotic rather than lytic bone lesions, demyelinating polyneuropathy, and a constellation of endocrinopathies including hypogonadism, thyroid dysfunction, and diabetes mellitus [2,3]. Patients often present with extravascular fluid overload, such as ascites, pleural effusions, and peripheral oedema, mediated by elevated VEGF levels [4,5]. VEGF overexpression is a characteristic feature of the syndrome and leads to vascular hyperpermeability, angiogenesis, and a prothrombotic state, contributing to complications such as Budd-Chiari syndrome, pulmonary hypertension, and thrombotic events [4,6,7]. The underlying pathophysiology involves a clonal PCD, most commonly secreting lambda light chains, which triggers a cascade of cytokine release, including VEGF, IL-6, and TNF-alpha, resulting in diverse systemic manifestations [4,6].

Early recognition of neuropathic presentations is crucial, as they may be preceded by systemic manifestations by several months to years [3,6]. Electrophysiology typically demonstrates symmetric sensorimotor demyelinating patterns, as observed in our patient. Early treatment can halt disease progression and improve functional outcomes [3,5].

Management of POEMS syndrome primarily focuses on suppression of the underlying plasma cell clone using targeted therapies.

In patients who are not candidates for transplantation or have diffuse disease, proteasome inhibitors (e.g., bortezomib), immunomodulatory drugs (e.g., lenalidomide or thalidomide), and monoclonal antibodies (e.g., daratumumab) are increasingly used [7,8]. These agents are typically combined with low-dose corticosteroids, such as dexamethasone, to optimise efficacy and tolerability. Recent case series indicate that daratumumab-containing regimens are effective in relapsed or refractory POEMS, leading to rapid haematologic responses and decreased VEGF levels [7]. Supportive care is also essential and includes management of fluid overload, anticoagulation for prothrombotic complications, physiotherapy for neuropathy, and endocrine replacement therapy [2,3]. VEGF can serve as a biomarker for disease activity, treatment response, and therapeutic decision-making [4]. Long-term follow-up is recommended, as relapse can occur, and early intervention strongly influences prognosis [3]. The multisystem nature of POEMS favors a multidisciplinary approach, involving haematology, neurology, endocrinology, hepatology, and rehabilitation services [5,9]. Complications such as Budd-Chiari syndrome, although rare, reflect the prothrombotic state induced by VEGF and cytokine imbalance and require prompt recognition and anticoagulation [9]. Prognosis has improved significantly over the past two decades due to advances in therapy and supportive care, with five-year survival rates exceeding 70%-80% in recent cohorts [5,7].

## Conclusions

POEMS syndrome should be suspected in cases of unexplained neuropathy accompanied by a multisystem presentation. Budd-Chiari syndrome may occur as a vascular complication due to thrombosis. Elevated VEGF levels are both diagnostic and predictive of disease activity. Investigations (including VEGF levels; full blood count; liver, renal, and thyroid function tests; skeletal surveys; follow-up imaging; and nerve conduction studies) should be monitored routinely. Regular follow-up is essential to assess treatment response, disease progression, and symptom improvement. Early targeted therapy is associated with favourable outcomes, although the long-term sequelae of neuropathy in POEMS remain uncertain. This case highlights the complex diagnostic overlap among haematology, neurology, and hepatology encountered in acute settings. It emphasises the importance of maintaining a broad differential diagnosis and considering rare, potentially life-threatening diseases in multisystem presentations. The key take-home messages are the importance of early recognition of POEMS syndrome and awareness of its potential vascular complications.
